# KIR3DS1/L1 and HLA-Bw4-80I are associated with HIV disease progression among HIV typical progressors and long-term nonprogressors

**DOI:** 10.1186/1471-2334-13-405

**Published:** 2013-09-02

**Authors:** Yongjun Jiang, Ou Chen, Chen Cui, Bin Zhao, Xiaoxu Han, Zining Zhang, Jing Liu, Junjie Xu, Qinghai Hu, Christina Liao, Hong Shang

**Affiliations:** 1 Key Laboratory of AIDS Immunology of Ministry of Health, Department of Laboratory Medicine, The First Affiliated Hospital, China Medical University, Shenyang 110001, P. R. China; 2 Collaborative Innovation Center for Diagnosis and Treatment of Infectious Diseases, Hangzhou, China; 3 Department of Laboratory Medicine, The Fourth Affiliated Hospital, China Medical University, Shenyang 110032, P. R. China

**Keywords:** Natural killer (NK) cells, Killer immunoglobulin-like receptors (KIRs), Human immunodeficiency virus (HIV), Long-term nonprogressor (LTNP), Typical progressor (TP)

## Abstract

**Background:**

Natural killer (NK) cells have emerged as pivotal players in innate immunity, especially in the defense against viral infections and tumors. Killer immunoglobulin-like receptors (KIRs) – an important recognition receptor expressed on the surface of NK cells – regulate the inhibition and/or activation of NK cells after interacting with human leukocyte antigen (HLA) class I ligands. Various KIR genes might impact the prognosis of many different diseases. The implications of KIR-HLA interaction in HIV disease progression remains poorly understood.

**Methods:**

Here, we studied KIR genotypes, mRNA levels, HLA genotypes, CD4^+^ T cell counts and viral loads in our cohort of Human Immunodeficiency Virus (HIV)-infected individuals, a group that includes HIV long-term nonprogressors (LTNPs) and typical progressors (TPs).

**Results:**

We found that the frequency of KIR3DS1/L1 heterozygotes with HLA-Bw4-80I gene was much higher in LTNPs than in TPs (*P* = 0.001) and that the KIR3DL1 homozygotes without HLA-Bw4-80I gene had higher viral loads and lower CD4^+^ T cell counts (*P* = 0.014 and *P* = 0.021, respectively). Our study also confirmed that homozygosity for the HLA-Bw6 allele was associated with rapid disease progression. In addition to the aforementioned results on the DNA level, we observed that higher level expression of KIR3DS1 mRNA was in LTNP group, and that higher level expression of KIR3DL1 mRNA was in TP group.

**Conclusions:**

Our data suggest that different KIR-HLA genotypes and different levels of transcripts associate with HIV disease progression.

## Background

Natural killer (NK) cells play a vital role in innate immune response because of their ability to kill virus-infected cells, to produce cytokines, and to communicate with the adaptive immune system. NK cell activation is controlled by complex interactions between activating or inhibitory receptors and their associated ligands. Of these receptors, the killer immunoglobulin-like receptors (KIRs), which interact with human leukocyte antigen (HLA) class I ligands on the surface of target cells, are the main receptors that recognize the presence or absence of antigens on cell surface [1,2].

The KIR region, located on chromosome 19q13.4, is highly polymorphic in humans, and the polygenic KIR gene complex codes for varying numbers of inhibitory and activating receptors [3]. To date, 14 KIR genes and two pseudogenes have been discovered. KIRs contain two or three external immunoglobulin-like domains (KIR2D, KIR3D) with either long (L) or short (S) cytoplasmic tails, corresponding to their function as inhibitory or activating receptors, respectively [1,4-6]. HLA class I genes, located on chromosome 6p21.3, are KIR ligands. The degree of NK cell inhibition and/or activation is regulated by interactions between KIR and HLA class I gene products [7]. Both KIR and HLA genes exhibit remarkable diversity and rapid evolution, suggesting that they are governed by pathogen-mediated selection and that they influence disease outcomes in individuals [8].

Indeed, several disease association studies have indicated that interactions between KIR and HLA class I ligands play a role in controlling infectious diseases, autoimmune/inflammatory disorders, cancers and reproduction diseases [1,3]. The KIR3DS1 gene and the HLA-Bw4-80I gene, have reported links to the slowing down of Human Immunodeficiency Virus (HIV) disease progression. However, some researchers have drawn different, even opposing, conclusions [9-14]. The relationship between the KIR3DL1 allele and HIV disease progression is also full of controversy. KIR3DL1, which segregates as an allele of the KIR3DS1 gene, is supposed to have an inhibitory function on NK cell activity. A study by Carr, W.H. et al., revealed that KIR3DL1 contributes to an inhibitory response when bound to HLA-Bw4 ligands [15]. Several studies also showed that the presence of inhibitory allele KIR3DL1 in combination with the HLA-B*57 s alleles that contain the Bw4-80I epitope had a highly protective effect against progression to AIDS [16,17].

Besides the above research, other KIRs had also been reported to be associated with HIV disease progression. Specifically, Gaudieri, S, et al., found that KIR2DS2/L2 was associated with more rapid decline in CD4^+^ T cells and a faster progression towards AIDS [13]. In addition, Soria, A. et al., found that the functional compound genotype HLA-C1(+)/KIR2DL3(+), was associated with reduced risk of immunological no responder status in treated HIV-infected individuals [18].

Long-term nonprogressors (LTNPs), who experience markedly slower disease progression and represent 2% to 5% of HIV-infected individuals [19], serve as an ideal model for studying the biological mechanisms of the slow progression to AIDS [20]. However, the KIR genes of LTNPs have rarely been studied and require further investigation.

Previous studies on the association between HIV disease progression and the interaction of KIRs and their ligands focused mainly on DNA data. However, studying these genes at the transcriptional level could provide more relevant information in protein function. Nevertheless, data related to relative quantitative mRNA expressions of KIR in HIV-infected individuals were seldom reported. One study reported that high levels of simian immunodeficiency virus (SIV) replication were associated with significant increases in KIR3DL mRNA levels among SIV-infected rhesus macaques [21]. The nature of the association between KIR mRNA and HIV disease progression is still unclear.

In this report, we explored the associations between HIV disease progression and the following factors: the KIR genes, the HLA genes, the combination of these genes, and mRNA levels in HIV-infected individuals.

## Methods

### Study population

A total of 132 HIV-seropositive individuals, including 40 selective LTNPs, were recruited for this study, all of whom were Chinese and ethnically Han, the predominant ethnic group in China. All HIV-seropositive individuals were grouped according to their CD4^+^ T cell counts. If their CD4^+^ T cell counts were above 500 cells/μl, we put them in the “high CD4^+^ T cell count” group. If their CD4^+^ T cell counts were below 500 cells/μl, we put them in the “low CD4^+^ T cell count” group. According to these criteria, 49 individuals belonged to the “high CD4^+^ T cell count” group and 83 individuals belonged to the “low CD4^+^ T cell count” group. The individuals were then grouped according to HIV viral loads. Of these individuals, 84 individuals who had viral loads more than 10^4^ copies/ml were placed in the “high viral load” group, and 48 individuals who had viral loads less than 10^4^ copies/ml were placed in the “low viral load” group. Finally, individuals were grouped according to HIV disease progression; the LTNP group was composed of 40 individuals who had no history of antiretroviral therapy and maintained CD4^+^ T cell counts above 500 cells/μl for more than 10 years of confirmed HIV infection. The TP group was composed of 83 individuals who also had no history of antiretroviral therapy and whose CD4^+^ T cell counts had dropped lower than 500 cells/μl. The KIR genotypes, HLA genotypes, CD4^+^ T cell counts and viral loads for all study participants were determined. The Research and Ethics Committee of The First Affiliated Hospital of China Medical University approved the study, and each studied individual gave written informed consent for participation in the study.

### KIR and HLA genotyping

Genomic DNA was extracted from whole blood samples of HIV-seropositive individuals using QIAamp DNA Mini Kits (Qiagen, Germany) according to the manufacturer’s instructions. KIR and HLA genotypings were done by performing PCR-SSP (polymerase chain reaction sequence-specified primer) using the KIR Genotyping SSP Kit (Tianjin Super Biotechnology Development Corporation, China) and Micro SSP™ Generic HLA Class I DNA Typing Tray (One Lambda, Inc, USA). KIR genotyping was performed using PCR amplification with primers specific for each locus (PCR-SSP). Internal control primers for a 588-bp conserved fragment of the human growth hormone (HGH) were also included in each PCR. Two sets of primers were used for each locus. The presence or absence of KIR genes was determined by comparing with the standard provided by the manufacturer.

### Analysis of nucleotides and amino acids of HLA-B

HLA-B genotyping was done by performing PCR-SSP. Genomic DNA from HIV-seropositive individuals was used as a template. Amplification primers Bx1 (5′-GGGAGGAGCGAGGGGACC(G/C)CAG-3′) and BINT3 (5′-GGAGGCCATCCCCGGCGACCTAT-3′) allowed amplification of an HLA-B-specific product of about 1000 base pairs. The purified HLA-B amplification product was used as a template for direct cycle-sequencing reactions, and HLA-Bw4-80I was characterized by the presence of isoleucine at position 80 of the second exon from the C- terminus.

### Determination of CD4^+^ T cell counts

CD4^+^ T cell counts were measured using a FACSCalibur flow cytometer (BD Bioscience, San Jose, CA, USA). A single-platform lyse-no-wash procedure was performed using TruCOUNT tubes and TriTEST anti-CD4-FITC/CD8-PE/CD3-PerCP reagents (BD, USA). TruCOUNT Control beads (low, median and high beads) were used to ensure the quality of the CD4^+^ T cell test.

### HIV viral load measurement

HIV viral loads in plasma were detected by performing RT-PCR using the COBAS Amplicor HIV Monitor 1.5 (RocheMolecular Systems, Branchbury, NJ, USA) with a detection limit of between 400 copies/ml and 7.5 × 10^5^ copies/ml.

### Relative quantitative analysis of KIR mRNA expression

RNA was isolated from peripheral blood mononuclear cells (PBMCs) using QIAamp RNA Mini Kits (Qiagen, Germany) and assayed for purity and concentration. Total RNA (1 μg) was converted to cDNA using the Improm-II™ Reverse Transcriptim System (Promege, USA). Specific primers for the KIR genes of interest were synthesized according to the reported method [22,23]. The glyceraldehyde-3-phosphate dehydrogenase (GADPH) gene was selected as a reference gene to control for different input RNA [24,25]. The primers (forward and reverse sequences) for the KIR3DS1 gene were 5′- CAGCGCTGTGGTGCCTCGC-3′ and 5′-CTGTGACCATGATCACCAT-3′ [22], for the KIR3DL1 gene were 5′-GGACATCGTGGTCACAGGTCC-3′ and 5′- CACTGAGGTCCCAATCAGAATG-3′ [25], for the GAPDH gene were, 5′-GGTGGTCTCCTCTGACTTCAACA-3′ and 5′-GTTGCTGTAGCCAAATTCGTTGT-3′. The specificity of these primer sets was confirmed by PCR and DNA sequencing (done by BGI China). A plasmid DNA standard was first constructed. Briefly, the KIR3DS1, KIR3DL1 and GADPH mRNAs were amplified by PCR from their respective cDNA fragments. The products were cloned into pMD 18-T vectors (Takara, Japan) The plasmid standard was serially diluted (1:10, 1:10^2^, 1:10^3^, 1:10^4^, 1:10^5^) and used for PCR. Real-time quantitative PCR amplification was performed using SYBR Premix Ex Taq™ (Takara, Japan). Cycling conditions were: 1 initial cycle, 30 seconds at 95°C; followed by 40 cycles, 30 seconds at 95°C; and 1 final cycle, 34 seconds at 60°C. To ensure specificity, dissociation curves were analyzed after each run. The relative expression of KIR mRNA was normalized to the expression of GAPDH in total RNA preparations.

### Statistical analysis

Differences in KIR and HLA frequencies between the various study groups were assessed by using the **χ**^**2**^ test for categorical variables. P values were calculated by using Yates’s correction test or Fisher’s exact test. KIR mRNA levels were compared between the groups by using non-parametric test analysis. Spearman’s rank correlation was used to perform the correlation analysis. All analyses were carried out using SPSS 17.0 software. P values < 0.05 were considered significant.

## Results

### Relationship between the frequencies of KIR genes and HIV disease progression

We screened 14 KIR genes and two psuedogenes for each participant in our study. The frequencies of individuals positive for each gene among the whole study population were calculated and listed in Additional file 1: Table S1. In our study population, we found that two alleles in the KIR gene cluster (KIR3DL3 and KIR3DL2) were present in 100% of participants. The frequencies of individuals with the KIR2DL4 gene or the KIR3DP1 gene were also approximately 100% in our pariticipants.

Firstly, we examined the associations between various KIR genes and the maintenance of CD4^+^ T cell counts. We found that the frequency of individuals with KIR3DS1 gene was significantly higher in the “high CD4^+^ T cell count” group than in the “low CD4^+^ T cell count” group (44.9% vs. 26.5%, *P* = 0.025; Table 1). However, the frequency of individuals with the KIR3DL1 gene was not different between the two groups (*P* = 0.266; Table 1). There were no statistical differences in the frequencies of individuals with other KIR genes between the “high CD4^+^ T cell count” group and the “low CD4^+^ T cell count” group (Table 1). In addition, we found that the KIR3DS1 allele frequency was higher in the “high CD4^+^ T cell count” group than in the “low CD4^+^ T cell count” group (*P* = 0.02; Table 2). Conversely, the KIR3DL1 allele frequency was lower in the “high CD4^+^ T cell count” group than in the “low CD4^+^ T cell count” group (*P* = 0.02; Table 2).

**Table 1:** **Distribution of KIR gene frequency**^**1 **^**among individuals in different groups**

**KIRgene**	^**2**^**LowCD4**	^**3**^**High CD4**	***P***	^**4**^**High VL**	^**5**^**Low VL**	***P***	^**6**^**TP**	^**7**^**LTNP**	***P***
	**(Total 83)**	**(Total 49)**		**(Total 84)**	**(Total 48)**		**(Total 83)**	**(Total 40)**	
	**n (%)**	**n (%)**		**n (%)**	**n (%)**		**n (%)**	**n (%)**	
KIR2DL1	80 (96.4)	47 (95.9)	0.615	81 (96.4)	46 (95.8)	0.601	80 (96.4)	40 (100.0)	0.304
KIR2DL2	43 (51.8)	32 (65.3)	0.091	46 (54.8)	29 (60.4)	0.328	43 (51.8)	26 (65.0)	0.117
KIR2DL3	81 (97.6)	46 (93.9)	0.266	82 (97.6)	45 (93.8)	0.253	81 (97.6)	40 (100.0)	0.454
KIR2DL4	82 (98.8)	49 (100.0)	0.629	83 (98.8)	47 (97.9)	0.597	82 (98.8)	40 (100.0)	0.675
KIR2DL5	35 (42.2)	22 (44.9)	0.450	36 (42.9)	21 (43.8)	0.532	35 (42.2)	18 (45.0)	0.458
KIR2DS1	34 (41.0)	26 (53.1)	0.122	36 (42.9)	24 (50.0)	0.270	34 (41.0)	21 (52.5)	0.156
KIR2DS2	23 (27.7)	12 (24.5)	0.424	19 (22.6)	16 (33.3)	0.128	23 (27.7)	9 (22.5)	0.350
KIR2DS3	13 (15.7)	11 (22.4)	0.227	15 (17.9)	9 (18.8)	0.537	13 (15.7)	11 (27.5)	0.097
KIR2DS4	81 (97.6)	46 (93.9)	0.266	82 (97.6)	45 (93.8)	0.253	81 (97.6)	37 (92.5)	0.194
KIR2DS5	26 (31.3)	18 (36.7)	0.326	27 (32.1)	17 (35.4)	0.422	26 (31.3)	14 (35.0)	0.417
KIR2DP1	80 (96.4)	47 (95.9)	0.615	81 (96.4)	46 (95.8)	0.601	80 (96.4)	40 (100.0)	0.304
KIR3DP1	83 (100.0)	48 (98.0)	0.371	84 (100.0)	47 (97.9)	0.364	83 (100.0)	39 (97.5)	0.325
KIR3DL1	81 (97.6)	46 (93.9)	0.266	82 (97.6)	45 (93.8)	0.253	81 (97.6)	37 (92.5)	0.194
KIR3DL2	83 (100.0)	49 (100.0)		84 (100.0)	48 (100.0)		83 (100.0)	40 (100.0)	
KIR3DL3	83 (100.0)	49 (100.0)		84 (100.0)	48 (100.0)		83 (100.0)	40 (100.0)	
KIR3DS1	22 (26.5)	22 (44.9)	**0.025**	25 (29.8)	19 (39.6)	0.169	22 (26.5)	18 (45.0)	**0.033**

^1^The frequency of individuals with at least one copy of the 16 KIR genes.

^2^Low CD4, “low CD4^+^ T cell count” group (CD4^+^ T cell counts ≥ 500 cells/μl); ^3^High CD4, “High CD4^+^ T cell count” group (CD4^+^ T cell counts<500 cells/μl); ^4^High VL, “high viral load” group (HIV viral load ≥ 10^4^ copies/ml); ^5^Low VL, “low viral load” group (HIV viral load<10^4^ copies/ml); ^6^TP, HIV typical progressors; ^7^LTNP, HIV long-term nonprogressors (9 individuals were excluded for the analysis based on LTNPs or TPs, because of not meeting the criteria of LTNPs or TPs).

n, the number of individuals with each KIR gene in each group; %, the percentage of individuals with each KIR gene in each group.

Data was calculated by from Yates’s correction or Fisher’s exact test.

Bold numbers indicated P value<0.05.

**Table 2:** The allele frequency of KIR3DS1 or KIR3DL1 between different groups

**KIR alleles**	^**1**^**Low CD4**	^**2**^**High CD4**	***P***	^**3**^**High VL**	^**4**^**Low VL**	***P***	^**5**^**TP**	^**6**^**LTNP**	***P***
	**n/166 (%)**	**n/98 (%)**		**n/168 (%)**	**n/96 (%)**		**n/166 (%)**	**n/80 (%)**	
KIR3DL1	142/166 (85.5)	73/98 (74.5)	**0.02**	141/168 (83.9)	74/96 (77.1)	0.11	142/166 (85.5)	59/80 (73.7)	**0.03**
KIR3DS1	24/166 (14.5)	25/98 (25.5)	**0.02**	27/168 (16.1)	22/96 (22.9)	0.11	24/166 (14.5)	21/80 (26.3)	**0.03**

Data are the frequencies of KIR alleles (assuming the pairs 3DS1/3DL1 are alleles of respective loci).

^1^Low CD4, “low CD4^+^ T cell count” group (CD4^+^ T cell counts<500 cells/μl); ^2^High CD4, “High CD4^+^ T cell count” group (CD4^+^ T cell counts ≥ 500 cells/μl); ^3^High VL, “high viral load” group (HIV viral load ≥ 10^4^ copies/ml); ^4^Low VL, “low viral load” group (HIV viral load<10^4^ copies/ml); ^5^TP, HIV typical progressors; ^6^LTNP, HIV long-term nonprogressors (9 individuals were excluded for the analysis based on LTNPs or TPs, because of not meeting the criteria of LTNPs or TPs).

Data was calculated by from Yates’s correction or Fisher’s exact test.

Bold numbers indicate P value<0.05.

Then, we investigated the associations between the KIR genes and HIV viral loads. There were no significant differences in the frequencies of individuals with the KIR3DS1 gene or the KIR3DL1 gene between the “high viral load” group and the “low viral load” group (29.8% vs. 39.6% for KIR3DS1, *P* = 0.169; 97.6% vs. 93.8% for KIR3DL1, *P* = 0.253; Table 1). In fact, there were no statistically significant differences in the frequencies of individuals with other KIR genes between the “low viral load” group and the “high viral load” group.

In addition to these results, we observed that the frequency of individuals with the KIR3DS1 gene was higher in LTNPs than in TPs (45.0% vs. 26.5%, *P* = 0.033; Table 1). However, there was no statistical difference in the frequency of individuals with the KIR3DL1 gene between TPs and LTNPs (97.6% vs. 92.5%, *P* = 0.194; Table 1). However, there were no statistical differences in the frequencies of individuals with other KIR genes between the LTNP group and the TP group either (Table 1). Additionally, we found that the KIR3DS1 allele frequency was higher in LTNPs than in TPs (*P* = 0.03; Table 2), and the KIR3DL1 allele frequency was lower in LTNPs than in TPs (*P* = 0.03; Table 2).

Finally, we analyzed the differences between different groups in the KIR3DS1/L1 genotypes (KIR3DS1 homozygotes, KIR3DS1/L1 heterozygotes and KIR3DL1 homozygotes). We found that the frequency of KIR3DL1 homozygotes (KIR3DL1/L1) was higher in the“low CD4^+^ T cell count” group than in the “high CD4^+^ T cell count” group (*P* = 0.025, Table 3). Similarly, the frequency of the KIR3DL1 homozygotes in TPs was higher than in LTNPs (*P* = 0.033, Table 3). However, there was no difference between the “high viral load” group and the “low viral load” group. We didn’t observe that the frequencies of KIR3DS1 homozygotes (KIR3DS1/S1) or KIR3DS1/L1 heterozygotes (KIR3DS1/L1) were different between groups (Table 3).

**Table 3:** Genotypic distribution of 3DS1/3DL1 and the ligand among individuals in different groups

**Allele genotype, ligand**	^**1**^**Low CD4**	^**2**^**High CD4**	***P***	^**3**^**High VL**	^**4**^**Low VL**	***P***	^**5**^**TP**	^**6**^**LTNP**	***P***
	**(Total 83)**	**(Total 49)**		**(Total 84)**	**(Total 48)**		**(Total 83)**	**(Total 40)**	
	**n (%)**	**n (%)**		**n (%)**	**n (%)**		**n (%)**	**n (%)**	
3DS1/3DL1	20 (24.1)	19 (38.8)	0.057	23 (27.4)	16 (33.3)	0.299	20 (24.1)	15 (37.5)	0.093
3DS1/3DS1	2 (2.4)	3 (6.1)	0.266	2 (2.4)	3 (6.3)	0.253	2 (2.4)	3 (7.5)	0.194
3DL1/3DL1	61 (73.5)	27 (55.1)	**0.025**	59 (70.2)	29 (60.4)	0.169	61 (73.5)	22 (55.0)	**0.033**
3DS1/?, with 80I	2 (2.4)	9 (18.4)	**0.002**	7 (8.3)	4 (8.3)	0.636	2 (2.4)	9 (22.5)	**0.001**
3DL1/?, with 80I	11 (13.3)	15 (30.6)	**0.015**	13 (15.5)	13 (27.1)	0.084	11 (13.3)	14 (35.0)	**0.006**
3DS1/3DS1, with 80I	0 (0.0)	0 (0.0)		0 (0.0)	0 (0.0)		0 (0.0)	0 (0.0)	
3DL1/3DL1, with 80I	9 (10.8)	6 (12.2)	0.508	6 (7.1)	9 (18.8)	**0.043**	9 (10.8)	5 (12.5)	0.501
3DS1/3DL1, with 80I	2 (2.4)	9 (18.4)	**0.002**	7 (8.3)	4 (8.3)	0.636	2 (2.4)	9 (22.5)	**0.001**
3DS1/?, no 80I	20 (24.1)	13 (26.5)	0.455	18 (21.4)	15 (31.2)	0.148	20 (24.1)	9 (22.5)	0.518
3DL1/?, no 80I	70 (84.3)	31 (63.3)	**0.006**	69 (82.1)	32 (66.7)	**0.037**	70 (84.3)	23 (57.5)	**0.002**
3DS1/3DS1, no 80I	2 (2.4)	3 (6.1)	0.266	2 (2.4)	3 (6.3)	0.253	2 (2.4)	3 (7.5)	0.194
3DL1/3DL1, no 80I	52 (62.7)	21 (42.9)	**0.021**	53 (63.1)	20 (41.7)	**0.014**	52 (62.7)	17 (42.5)	**0.028**
3DS1/3DL1, no 80I	18 (21.7)	10 (20.4)	0.523	16 (19.1)	12 (25.0)	0.277	18 (21.7)	6 (15.0)	0.267
3DS1/?, with 80 T	1 (1.2)	0 (0.0)	0.629	1 (1.2)	0 (0.0)	0.636	1 (1.2)	0 (0.0)	0.675
3DL1/?, with 80 T	7 (8.4)	1 (2.0)	0.132	5 (6.0)	3 (6.3)	0.609	7 (8.4)	1 (2.5)	0.2
3DL1/3DL1, with 80 T	6 (7.2)	1 (2.0)	0.192	4 (4.8)	3 (6.3)	0.501	6 (7.2)	1 (2.5)	0.271
3DS1/3DL1, with 80 T	1 (1.2)	0 (0.0)	0.629	1 (1.2)	0 (0.0)	0.636	1 (1.2)	0 (0.0)	0.675
3DS1/3DS1, with 80 T	0 (0.0)	0 (0.0)		0 (0.0)	0 (0.0)		0 (0.0)	0 (0.0)	
3DS1/?, no 80 T	21 (25.3)	22 (44.9)	**0.017**	24 (28.6)	19 (39.6)	0.135	21 (25.3)	18 (45.0)	**0.024**
3DL1/?, no 80 T	74 (89.2)	45 (91.8)	0.431	77 (91.7)	42 (87.5)	0.314	74 (89.2)	36 (90.0)	0.579
3DL1/3DL1, no 80 T	55 (66.3)	26 (53.1)	0.094	55 (65.5)	26 (54.2)	0.136	55 (66.3)	21 (52.5)	0.102
3DS1/3DL1, no 80 T	19 (22.9)	19 (53.1)	**0.041**	22 (26.2)	16 (33.3)	0.25	19 (22.9)	15 (37.5)	0.071
3DS1/3DS1, no 80 T	2 (2.4)	3 (6.1)	0.266	2 (2.4)	3 (6.3)	0.253	2 (2.4)	3 (7.5)	0.194

^1^Low CD4, “low CD4^+^ T cell count” group (CD4^+^ T cell counts<500 cells/μl); ^2^High CD4, “High CD4^+^ T cell count” group (CD4^+^ T cell counts ≥ 500 cells/μl); ^3^High VL, “high viral load” group (HIV viral load ≥ 10^4^ copies/ml); ^4^Low VL, “low viral load” group (HIV viral load<10^4^ copies/ml); ^5^TP, HIV typical progressors; ^6^LTNP, HIV long-term nonprogressors (9 individuals were excluded for the analysis based on LTNPs or TPs, because of not meeting the criterions of LTNPs or TPs).

3DS1/L1, KIR3DS1/L1 heterozygotes; 3DS1/S1, 3DS1 homozygotes; 3DL1/L1, KIR3DL1 homozygotes.

3DS1/?, individuals with at least one copy of KIR3DS1 gene; 3DL1/?, individuals with at least one copy of KIR3DL1 gene.

80I, HLA-Bw4-80I; 80 T, HLA-Bw4-80 T.

n, the number of individuals with each genotype in each group; %, the percentage of individuals with each genotype in each group

Data was calculated by from Yates’s correction or Fisher’s exact test.

Bold numbers indicate P value<0.05.

These data demonstrate that KIR3DS1 gene is associated with higher CD4^+^ T cell counts and might delay the disease progression.

### Association of the HLA-B gene with HIV disease progression

**Figure 1:**
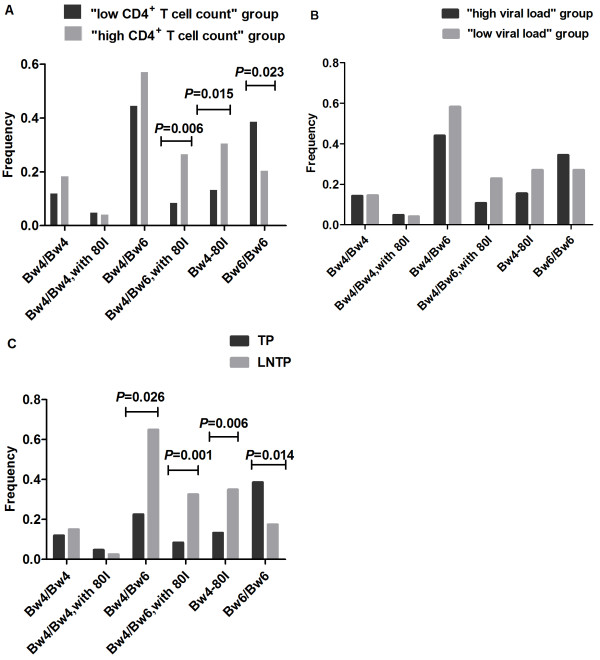
**Association of HLA-B gene with HIV disease progression.** Shown are results for the comparisons of the frequency of individuals in various study groups carrying a particular gene. **(A)** HLA-B gene frequencies among the “high CD4^+^ T cell count” group (light grey) and the “low CD4^+^ T cell count” group (dark grey) are shown. The data show that subjects who had at least one copy of the HLA-Bw4-80I gene had higher CD4^+^ T cell counts (*P* = 0.015). However, HLA-Bw6 homozygotes had lower CD4^+^ T cell counts (*P* = 0.023). **(B)** HLA-B gene frequencies among the “high viral load” group (light grey) and the “low viral load” group (dark grey) are shown. **(C)** HLA-B allele frequencies among TPs (light grey) and LTNPs (dark grey) are shown. The data show that the proportion of people who had at least one copy of the HLA-Bw4-80I gene was higher among LTNPs than among TPs (*P* = 0.006). However, the proportion of HLA-Bw6 homozygotes was significantly lower among LTNPs than among TPs (*P* = 0.014).

After analyzing the relationship between the KIR gene frequency and HIV disease progression, we subsequently divided our study participants according to their Bw4 or Bw6 epitopes, and analyzed the association between the HLA-B genes and HIV disease progression. We found that people with at least one copy of the HLA-Bw4-80I gene had higher CD4^+^ T cell counts (*P* = 0.015; Figure 1A). The proportion of Bw4/Bw6 heterozygous individuals with Bw4-80I was much higher in the “high CD4^+^ T cell count” group than in the “low CD4^+^ T cell count” group (*P* = 0.006; Figure 1A). However, we did not find any statistical differences in HLA-B gene frequencies between the “high viral load” group and the “low viral load” group (*P* > 0.05; Figure 1B). We found that the proportion of people who had at least one copy of HLA-Bw4-80I was higher among LTNPs (*P* = 0.006; Figure 1C). Moreover, the proportion of Bw4/Bw6 heterozygous individuals with Bw4-80I was also significantly higher among LTNPs, as compared with TPs (*P* =0.001; Figure 1C). Furthermore, we found that the proportion of HLA-Bw6 homozygotes was significantly lower among LTNPs (*P* = 0.014; Figure 1C).

Our results indicate that HLA-Bw4-80I might be associated with slower disease progression and HIV-infected individuals who are homozygous for HLA-Bw6 might experience faster disease progression.

### Association of the combination of KIR3DS1/3DL1 and HLA-Bw4-80I genes with disease progression

We were interested in determining whether the combination of the KIR3DS1/L1 genotype and the HLA-Bw4-80I gene had any association with HIV disease progression. Firstly, we analyzed the frequency of individuals with the KIR3DS1/L1 genotype and HLA-Bw4-80I gene among TPs and LTNPs. We found that the frequency of individuals with at least one copy of KIR3DS1 in the presence of HLA-Bw4-80I (3DS1/?, with 80I; Table 3) was notably higher in LTNPs than in TPs (*P* = 0.001; Table 3), and the frequency of KIR3DS1/L1 heterozygotes with HLA-Bw4-80I (3DS1/L1, with 80I; Table 3) was also higher in LTNPs than in TPs (*P* = 0.001; Table 3). Meanwhile, we also found that the frequency of individuals with at least one copy of KIR3DL1 in the presence of HLA-Bw4-80I (3DL1/?, with 80I; Table 3) was higher in LTNPs than in TPs (*P* = 0.006; Table 3), and conversely, the frequency of individuals with at least one copy of KIR3DL1 in absence of HLA-Bw4-80I (3DL1/?, no 80I; Table 3) was lower in LTNPs than in TPs (*P* = 0.002; Table 3). Moreover, the frequency of KIR3DL1 homozygotes without HLA-Bw4-80I (3DL1/L1, no 80I; Table 3) was much lower in LTNPs than in TPs (*P* = 0.028; Table 3).

Given that the combination of the KIR3DS1/3DL1 and HLA-Bw4-80I genes might be predictive of a patient’s ability to manage HIV replication and CD4^+^ T cell counts, we subsequently analyzed the association between the genotypes and HIV viral loads or CD4^+^ T cell counts. We found that the frequencies of individuals with at least one copy of KIR3DS1 in the presence of HLA-Bw4-80I (3DS1/?, with 80I; Table 3) was more common in the “high CD4^+^ T cell count” group than in the “low CD4^+^ T cell count” group (*P* = 0.002; Table 3). In addition, the frequency of individuals with at least one copy of KIR3DL1 in the presence of HLA-Bw4-80I (3DL1/?, with 80I; Table 3) was also more common in the “high CD4^+^ T cell count” group than in the “low CD4^+^ T cell count” group (*P* = 0.015; Table 3). However, we observed that the individuals with at least one copy of KIR3DL1in absence of HLA-Bw4-80I (3DL1/?, no 80I ) had lower CD4^+^ T cell counts and higher HIV viral loads (*P* = 0.006 and *P* = 0.037, respectively; Table 3). Similarly, KIR3DL1/L1 homozygotes without HLA-Bw4-80I (3DL1/L1, no 80I) also had lower CD4^+^ T cell counts and higher HIV viral loads (*P* = 0.021 and *P* = 0.014, respectively; Table 3).

Our analyses above were based on categorizations of CD4^+^ T cell counts, HIV viral loads and disease progression to compare the frequency of subjects with positive genes or alleles. The next analyses compared CD4^+^ T cell counts and HIV viral loads between different three groups classified as 3DS1^+^80I^+^, 3DS1^+^80I^-^ and the 3DS1^-^80I^+^. The individuals with at least one copy of KIR3DS1 in presence of HLA-Bw4-80I were in the “3DS1^+^80I^+^” group. The individuals with at least one copy of KIR3DS1 in absence of HLA-Bw4-80I were in the “3DS1^+^80I^-^” group, and KIR3DL1 homozygotes with HLA-Bw4-80I were in the “3DS1^-^80I^+^” group. We found that CD4^+^ T cell counts were much higher in the “3DS1^+^80I^+^” group than in the “3DS1^+^80I^-^” group (*P* = 0.041). However, there was no significant difference between the CD4^+^ T cell counts of the “3DS1^+^80I^+^” group and the “3DS1^-^80I^+^” group (*P* = 0.312). We didn’t find any statistical difference between the viral loads of the “3DS1^+^80I^+^” group and the “3DS1^+^80I^-^” group (*P* = 0.524). Neither was there a significant difference between the viral loads of the “3DS1^+^80I^+^” group and the “3DS1^-^80I^+^” group (*P* = 0.087) (data not shown). Altogether, these results demonstrate that the KIR3DS1 and HLA-Bw4-80I combined genotype is associated with slow HIV disease progression and higher CD4^+^ T cell counts.

In addition, we also analyzed the combination of KIR3DS1/L1 with or without HLA-Bw4-80 T among the different groups. There were no between group differences in the frequency of the KIR3DS1/L1 with HLA-Bw4-80 T combined genotype for comparisons based on CD4^+^ T cell counts, HIV viral load or disease progression. The frequency of individuals with at least one copy of KIR3DS1 in the absence of HLA-Bw4-80 T (3DS1/?, no 80 T; Table 3) was higher in LTNPs compared to TPs (*P* = 0.024;Table 3), and higher in the “high CD4^+^ T cell count” group compared to the “low CD4^+^ T cell count” group (*P* = 0.017;Table 3). We also found that the KIR3DS1/L1 heterozygotes without HLA-Bw4-80 T (3DS1/L1, no 80 T) was higher in the “high CD4^+^ T cell count” group compared to the “low CD4^+^ T cell count” group (*P* = 0.041;Table 3).

### KIR3DS1 and KIR3DL1 mRNA levels are associated with HIV disease progression

**Figure 2:**
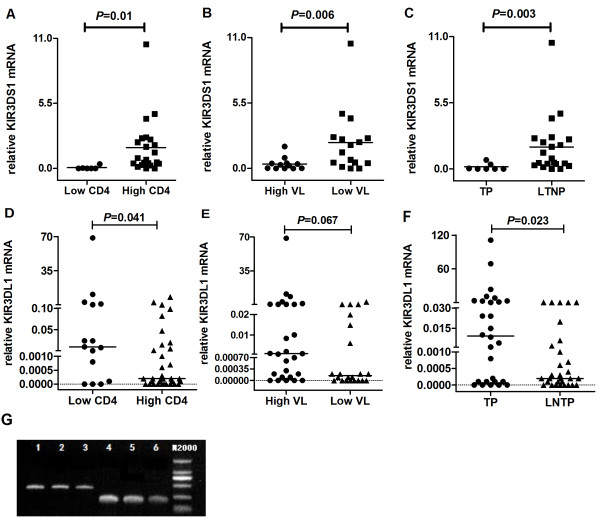
**KIR3DS1 and KIR3DL1 mRNA levels of different groups.** Shown are KIR transcript levels reflecting the relative expression of KIR3DS1 and KIR3DL1 mRNA normalized to GAPDH mRNA in total RNA preparations. Reported values are ratios of KIR and GAPDH concentrations. The lines denoted the medians. **(A)** KIR3DS1 mRNA levels of the “high CD4^+^ T cell count” group experienced a significant increase compared to KIR3DS1 mRNA levels in the “low CD4^+^ T cell count” group (*P* = 0.01). **(B)** KIR3DS1 mRNA levels of the “high viral load” group experienced a significant decrease compared to those in the “low viral load” group (*P* = 0.006). **(C)** Levels of KIR3DS1 mRNA in TPs were significantly lower than in LTNPs (*P* = 0.003). **(D)** KIR3DL1 mRNA levels in the “high CD4^+^ T cell count” group experienced a significant decrease compared to those in the “low CD4^+^ T cell count” group (*P* = 0.041). **(E)** There was no statistical difference between levels of KIR3DL1 mRNA in the “high viral load” group and the “low viral load” group (*P* = 0.067). **(F)** KIR3DL1 mRNA levels of TPs were significantly higher than those of LTNPs (*P* = 0.023). **(G)** The electrophoretogram was shown to demonstrate the RT-PCR specificity. The PCR reaction only generates a single band. Lane 1, 2 and 3 were for KIR3DL1, and lane 4, 5 and 6 were for KIR3DS1 using different primer concentration.

Our data suggest that the KIR3DS1/3DL1 gene is associated with HIV disease progression. To determine whether levels of KIR3DS1/KIR3DL1 mRNA impact HIV disease progression, we carried out relative quantitative analysis of KIR mRNA expression to compare the transcript levels of the KIR3DS1/KIR3DL1 gene among different groups. We observed that the KIR3DS1 mRNA levels were significantly higher in the “high CD4^+^ T cell count” group, the LNTP group, and the “low viral load” group (*P* = 0.01, *P* = 0.003 and *P* = 0.006, respectively; Figure 2A, 2B and 2C). Thus, these results suggest that individuals who have more KIR3DS1 mRNA may experience slower HIV disease progression.

We found different results regarding KIR3DL1, whose mRNA levels were significantly lower in the “high CD4^+^ T cell count” group than in the “low CD4^+^ T cell count” group (*P* = 0.041; Figure 2D). However, we found no significant difference between KIR3DL1 mRNA levels in the “low viral load” group and the “high viral load” group (*P* = 0.067; Figure 2E), but the mRNA levels were lower among LTNPs than among TPs (*P* = 0.023; Figure 2F). Together, these results indicate that mRNA levels of the KIR3DL1 gene in HIV-infected individuals may be associated with disease progression.

### Correlation between KIR3DS1 mRNA levels and CD4^+^ T cell counts or viral loads in HIV-infected individuals

**Figure 3:**
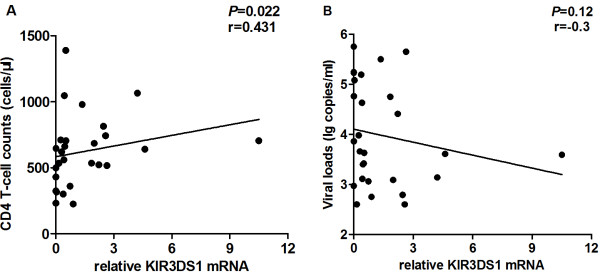
**Correlation between KIR3DS1 mRNA levels and CD4**^**+ **^**T cell counts and viral loads among HIV infected individuals. (A)** KIR3DS1 mRNA levels positively correlated with CD4^+^ T cell counts (*P* = 0.022, r = 0.431). **(B)** KIR3DS1 mRNA levels and viral loads shared no correlation (*P* = 0.12, r = −0.3). *P*-values < 0.05 were considered statistically significant.

Our results show that KIR3DS1 mRNA levels are positively correlated with CD4^+^ T cell counts (*P* = 0.022, r = 0.431; Figure 3A). This correlation indicates that individuals who have higher transcript quantities of KIR3DS1 have higher CD4^+^ T cell counts. However, there was no correlation between KIR3DS1 mRNA levels and viral loads (*P* = 0.12, r = −0.3; Figure 3B).

## Discussion

The KIR gene cluster codes for ligands of HLA class I molecules. Population-level data suggest the co-evolution of KIR and HLA gene clusters, which are functionally related but unlinked. However, the KIR genes evolve more rapidly than the HLA class I ligands [23]. Thus, we focused more on the association between the two gene clusters and HIV disease progression. By joint analysis of the two gene clusters together with HIV disease progression, we attempted to understand why some HIV-infected Chinese experienced fast disease progression while others experienced slow disease progression.

In our study, we observed that KIR3DS1 was associated with slower HIV disease progression in LTNPs and with the maintenance of CD4^+^ T cell counts. Other reports have also shown that KIR3DS1 independently associates with higher CD4^+^ T cell counts but does not have an effect on viral loads [12]. We speculated that KIR3DS1 might be associated with high NK cell function and result in slower disease progression. One study reported that NK cells in individuals who have the KIR3DS1^+^ allele produced more Interferon-γ (IFN-γ) compared to NK cells in individuals without KIR3DS1 [26]. Several studies also reported that activated human NK cells could enhance the function of CD4^+^ T cells and promote differentiation of Th1 cells by secreting IFN-γ [27]. Thus, we speculated that KIR3DS1, as an activating receptor, might enhance NK cell activity and help maintain the number of CD4^+^ T cells.

HLA-Bw4-80I, the ligand for the KIR3DL1 receptor and the putative ligand for the KIR3DS1 receptor, has been reported to be associated with HIV disease progression. Flores-Villanueva, et al. reported that, among Caucasians, homozygosity for HLA-Bw4 was strongly and independently associated with the ability to remain AIDS-free and maintain normal CD4^+^ T cell counts [28]. However, their analysis was specific to TPs and did not cover LTNPs. Conversely, in our study, we analyzed Chinese TPs and LTNPs. Our results showed that the proportion of HLA-Bw4 homozygotes is quite low among the Chinese population so there was no statistically significant difference in the frequency between LTNPs and TPs. But the frequency of heterozygosity for Bw4/Bw6 was much higher in LTNPs than in TPs. We also found that individuals who had at least one copy of HLA-Bw4-80I had higher CD4^+^ T counts and slower disease progression and tended to have lower HIV viral loads. The potential benefit of the HLA-Bw4 allele was related to the specificity of this ligand for NK cell inhibitory receptor KIR3DL1 [29]. The presentation of HIV antigens by HLA-Bw4 alleles might relieve the inhibition of NK activity, resulting in enhanced lysis of infected cells by NK cells, which could thereby influence HIV viremia and the subsequent progression towards AIDS[28-30]. Meanwhile, HLA-Bw4 might have greater binding affinity for the HIV antigen and induce a more effective immune response than HLA-Bw6 alleles [26]. In agreement with other studies [31], we also observed that HLA-Bw6 homozygosity was associated with rapid disease progression. In this study, we also analyzed the association between the combination of the KIR3DS1/KIR3DL1 and HLA-Bw4-80I genes with HIV disease progression. We found that KIR3DS1 together with HLA-Bw4-80I contribute to slower disease progression. We also observed that the absence of KIR3DS1 and HLA-Bw4-80I was associated with lower CD4^+^ T cell counts and higher viral loads. The mechanism of how this genetic combination affects HIV disease progression remains unclear [17,32]. HLA-B*57 is associated with slow disease progression and viral load control [14]. We did not find any subject with this allele in our study. Therefore, the association of KIR3DS1 and HLA-Bw4-80I with HIV disease course was not due to the influence HLA-B*57 may have on disease course in our study. KIR3DS1 activation might enhance NK cell activity and improved immune response. Recently, one functional association study reported that NK cells that were KIR3DS1^+^ inhibited HIV replication in HLA-Bw4-80I^+^ T cells [11]. These findings are consistent with our results at the functional level.

Conversely, we found that KIR3DL1 interaction with HLA-Bw4-80I was associated with slower CD4^+^ T cell count decline. The reason might be that the presence of both the inhibitory receptor KIR3DL1 and its HLA class I ligand HLA-Bw4-80I enhances NK cell responsiveness through a process known as NK cell “education” [33,34]. It seems that KIR3DL1^+^ NK cells exhibit a stronger response in the destruction of autologous HIV-infected cells when the inhibitory receptors are not bound to their ligands compared with KIR3DL1^-^ NK cells [35].

At the DNA level, we observed the association between KIR genes, especially KIR3DS1 and KIR3DL1, and HIV disease progression. We also identified an association between HIV disease progression and the levels of KIR3DS1/KIR3DL1 mRNA transcripts. Few studies were found related to quantitative mRNA expressions of the KIR gene cluster in HIV-infected individuals. Bostik, P. et al., reported that high levels of simian immunodeficiency virus (SIV) replication were associated with significant increases in KIR3DL1 mRNA levels among SIV-infected rhesus macaques [21]. Alter, G. et al. noted that levels of mRNA transcripts from activating KIRs were elevated in acute HIV infection and that levels of mRNA transcripts from inhibitory KIRs were elevated in chronic HIV infection [24]. In this study, we investigated the association between the mRNA expressions of KIR3DS1 or KIR3DL1 and HIV disease progression. We noted that individuals who had higher levels of KIR3DS1 mRNA had higher CD4^+^ T cell counts and lower viral loads. Conversely, those expressing more KIR3DL1 mRNA had faster disease progression and lower CD4^+^ T cell counts. It should be pointed out that one of the limitations of the results presented in this study is that although multiple analyses were conducted none were subjected to a Bonferonni correction.

## Conclusions

We demonstrated that the KIR3DS1 and KIR3DL1 gene with HLA-Bw4-80I gene have an association with the maintenance of CD4^+^ T cell count and slowing HIV disease progression. Our study also confirmed that homozygosity for the HLA-Bw6 allele was associated with rapid disease progression. We observed that high expression of KIR3DS1 mRNA was associated with slower disease progression, and high expression of KIR3DL1 mRNA was associated with rapid disease progression.

## Abbreviations

NK cells: Natural killer cells; KIR: Killer immunoglobulin-like receptor; KIR3DL1: Killer immunoglobulin-like receptor, three domains, long cytoplasmic tail 1; KIR3DS1: Killer immunoglobulin-like receptor, three domains, short cytoplasmic tail 1; HLA: Human leukocyte antigen; HIV: Human Immunodeficiency Virus; AIDS: Acquired immune deficiency syndrome; LTNP: Long-term nonprogressor; TP: Typical progressor.

## Competing interests

The authors declare that they have no competing interests.

## Authors’ contributions

YJ designed the experiments, analyzed the data and wrote the manuscript. YJ and OC carried out part of KIR genotyping and participated in the analysis of nucleotides and amino acids of HLA-B. CC and CL carried out part of KIR genotyping and drafted the manuscript. BZ carried out HLA-B genotyping and participated in the analysis of nucleotides and amino acids of HLA-B. XH participated in HIV viral load measurement. YJ and ZZ carried out determination of CD4^+^ T cell counts. JL, JX and QH were the clinical doctors who helped subject recruitment and carried out the epidemiological study. HS designed the study with YJ, verified and helped with interpretation of the data, and contributed to the writing. All authors read and approved the final manuscript.

## Pre-publication history

The pre-publication history for this paper can be accessed here:

http://www.biomedcentral.com/1471-2334/13/405/prepub

## Supplementary Material

Additional file 1: Table S1 KIR genotypes, HLA genotypes, CD4^+^ T cell counts and viral loads of study population. The table reports the genotypes and frequencies of KIR genes, HLA genotypes, CD4^+^ T cell counts and viral loads among our study population. Click here for file
